# Supplementary material for: Role and Regulation of ACC Deaminase Gene in Sinorhizobium meliloti: Is It a Symbiotic, Rhizospheric or Endophytic Gene?
Source: Front Genet. 2017 Jan 30;8:6. doi: 10.3389/fgene.2017.00006 (PMC5276845; doi:10.3389/fgene.2017.00006)
Supplement: Supplementary file 2 [file DataSheet2.DOCX]

Supplementary Material

Is ACC deaminase gene of *Sinorhizobium meliloti* a symbiotic or rhizospheric/endophytic gene?

**A. Checcucci, E. Azzarello, M. Bazzicalupo, A. De Carlo, G. Emiliani, S. Mancuso, G. Spini, C. Viti, A. Mengoni ***

*** Correspondence:** Corresponding Author: mail: [alessio.mengoni@unifi.it](mailto:alessio.mengoni@unifi.it)

# Supplementary Figures

## Supplementary Figures S1


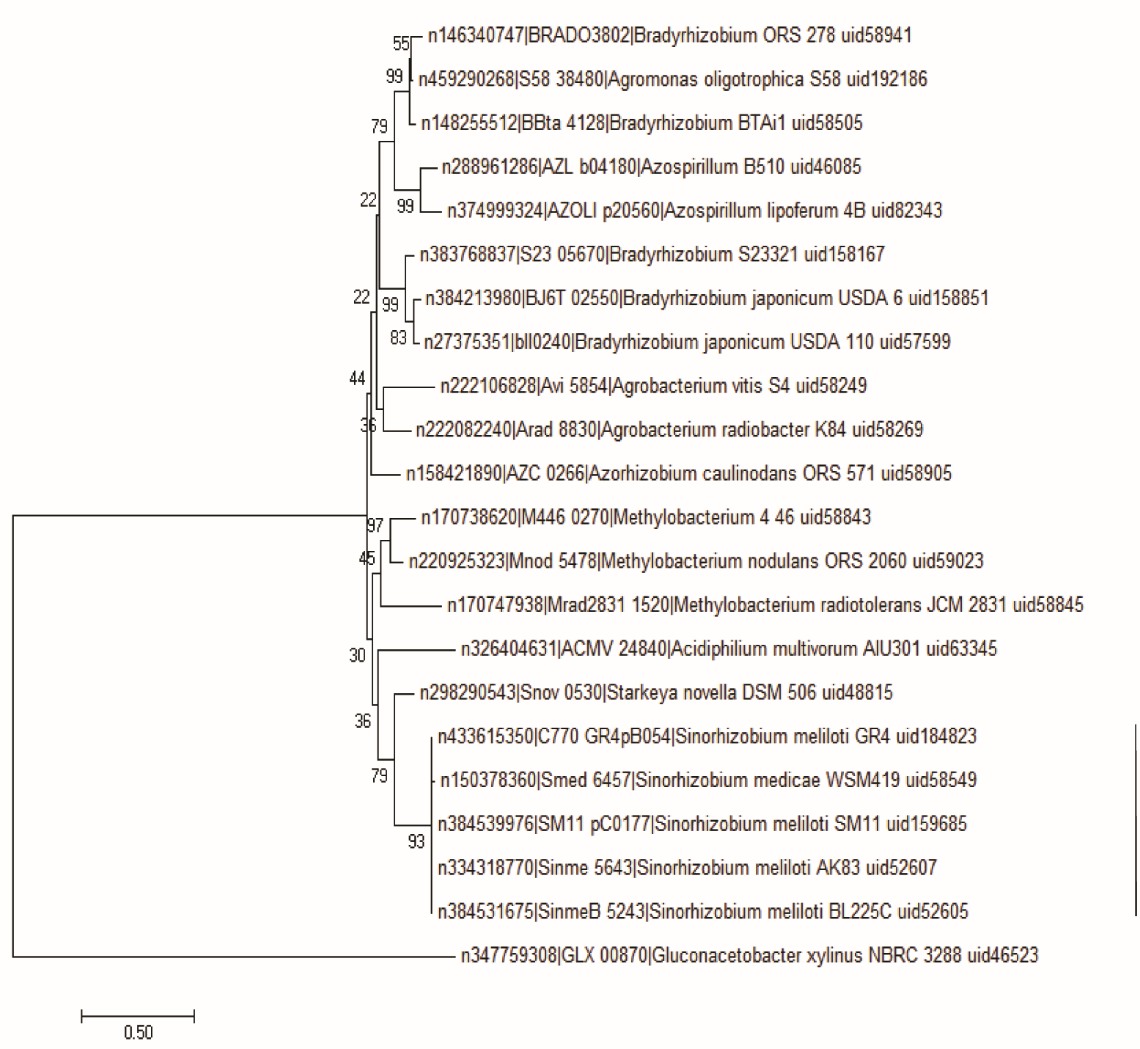


**Supplementary Figure S1.** **Phylogenetic pattern of *acdR* in acdS+ rhizobia.** Maximum Likelihood phylogenetic reconstruction of *acdR* sequences. For both *acdS* and *acdR* phylogenetic trees, numbers at nodes indicate bootstrap values after 500 random replicates. The GTR+G model has been chosen for the reconstructions after model test evaluation (Supplementary Data 1).

# Supplementary Tables

## Supplementary Table S1

**Supplementary Table S1**. **Wild type *S. meliloti* strains used in this work**. The name, origin of isolation and *acdS* presence (+, present; -, not present) are reported.

| **Strain name** | **Presence of *acdS*** | **Geographical origin** | **Host plant** | **Reference** |
| --- | --- | --- | --- | --- |
| 1A42 | - | Iran | *M. sativa* | Talebi Bedaf et al. 2008 |
| 1B23 | - | Iran | *M. sativa* | Talebi Bedaf et al. 2008 |
| 1B24 | - | Iran | *M. sativa* | Talebi Bedaf et al. 2008 |
| 1B54 | - | Iran | *M. sativa* | Talebi Bedaf et al. 2008 |
| 1B63 | - | Iran | *M. sativa* | Talebi Bedaf et al. 2008 |
| 1C54 | - | Iran | *M. sativa* | Talebi Bedaf et al. 2008 |
| 2A31 | - | Iran | *M. sativa* | Talebi Bedaf et al. 2008 |
| 2B13 | + | Iran | *M. sativa* | Talebi Bedaf et al. 2008 |
| 2C31 | - | Iran | *M. sativa* | Talebi Bedaf et al. 2008 |
| 3A11 | - | Iran | *M. sativa* | Talebi Bedaf et al. 2008 |
| 3B12 | - | Iran | *M. sativa* | Talebi Bedaf et al. 2008 |
| 3B13 | + | Iran | *M. sativa* | Talebi Bedaf et al. 2008 |
| 3B22 | - | Iran | *M. sativa* | Talebi Bedaf et al. 2008 |
| 3C21 | - | Iran | *M. sativa* | Talebi Bedaf et al. 2008 |
| 4A42 | - | Iran | *M. sativa* | Talebi Bedaf et al. 2008 |
| 4B41 | - | Iran | *M. sativa* | Talebi Bedaf et al. 2008 |
| 4C23 | - | Iran | *M. sativa* | Talebi Bedaf et al. 2008 |
| 5A14 | - | Iran | *M. sativa* | Talebi Bedaf et al. 2008 |
| 5B12 | - | Iran | *M. sativa* | Talebi Bedaf et al. 2008 |
| 5B44 | - | Iran | *M. sativa* | Talebi Bedaf et al. 2008 |
| 5C23 | + | Iran | *M. sativa* | Talebi Bedaf et al. 2008 |
| 5M15 | - | Iran | *M. sativa* | Talebi Bedaf et al. 2008 |
| 6A23 | - | Iran | *M. sativa* | Talebi Bedaf et al. 2008 |
| 6B43 | - | Iran | *M. sativa* | Talebi Bedaf et al. 2008 |
| 7B22 | - | Iran | *M. sativa* | Talebi Bedaf et al. 2008 |
| 7B31 | + | Iran | *M. sativa* | Talebi Bedaf et al. 2008 |
| 8A21 | - | Iran | *M. sativa* | Talebi Bedaf et al. 2008 |
| 8B53 | - | Iran | *M. sativa* | Talebi Bedaf et al. 2008 |
| 8B63 | - | Iran | *M. sativa* | Talebi Bedaf et al. 2008 |
| AE601M | - | Italy | *M. sativa* | Carelli et al. 2000 |
| AE608B | + | Italy | *M. sativa* | Carelli et al. 2000 |
| AE608B | - | Italy | *M. sativa* | Carelli et al. 2000 |
| AE608B | - | Italy | *M. sativa* | Carelli et al. 2000 |
| AE608D | - | Italy | *M. sativa* | Carelli et al. 2000 |
| AE608H | - | Italy | *M. sativa* | Carelli et al. 2000 |
| AK02 | - | Kazakhstan | *M. trautvetteri* | Roumiantseva et al 2014 |
| AK09 | - | Kazakhstan | *M. trautvetteri* | Roumiantseva et al 2014 |
| AK10 | - | Kazakhstan | *M. trautvetteri* | Roumiantseva et al 2014 |
| AK100 | - | Kazakhstan | *Melilotus sp.* | Roumiantseva et al 2014 |
| AK103 | + | Kazakhstan | *Melilotus sp.* | Roumiantseva et al 2014 |
| AK11 | - | Kazakhstan | *M.falcata* | Roumiantseva et al 2014 |
| AK111 | - | Kazakhstan | *M. trautvetteri* | Roumiantseva et al 2014 |
| AK120 | - | Kazakhstan | *M. trautvetteri* | Roumiantseva et al 2014 |
| AK132 | - | Kazakhstan | *M.varia* | Roumiantseva et al 2014 |
| AK133 | - | Kazakhstan | *M. varia* | Roumiantseva et al 2014 |
| AK135 | - | Kazakhstan | *M. trautvetteri* | Roumiantseva et al 2014 |
| AK138 | - | Kazakhstan | *M. trautvetteri* | Roumiantseva et al 2014 |
| AK140 | - | Kazakhstan | *M. trautvetteri* | Roumiantseva et al 2014 |
| AK152 | - | Kazakhstan | *M.falcata* | Roumiantseva et al 2014 |
| AK155 | - | Kazakhstan | *M. trautvetteri* | Roumiantseva et al 2014 |
| AK158 | - | Kazakhstan | *M. trautvetteri* | Roumiantseva et al 2014 |
| AK17 | + | Kazakhstan | *M. trautvetteri* | Roumiantseva et al 2014 |
| AK174 | - | Kazakhstan | *Trigonella sp.* | Roumiantseva et al 2014 |
| AK200 | - | Kazakhstan | *M.falcata* | Roumiantseva et al 2014 |
| AK201 | - | Kazakhstan | *M. trautvetteri* | Roumiantseva et al 2014 |
| AK21 | + | Kazakhstan | *M. trautvetteri* | Roumiantseva et al 2014 |
| AK27 | - | Kazakhstan | *M. trautvetteri* | Roumiantseva et al 2014 |
| AK33 | - | Kazakhstan | *Trigonella sp.* | Roumiantseva et al 2014 |
| AK34 | - | Kazakhstan | *Trigonella sp.* | Roumiantseva et al 2014 |
| AK37 | - | Kazakhstan | *Trigonella sp.* | Roumiantseva et al 2014 |
| AK43 | - | Kazakhstan | *M.varia* | Roumiantseva et al 2014 |
| AK48 | - | Kazakhstan | *M. trautvetteri* | Roumiantseva et al 2014 |
| AK49 | - | Kazakhstan | *M. trautvetteri* | Roumiantseva et al 2014 |
| AK50 | - | Kazakhstan | *M.varia* | Roumiantseva et al 2014 |
| AK52 | - | Kazakhstan | *M.varia* | Roumiantseva et al 2014 |
| AK54 | - | Kazakhstan | *M.falcata* | Roumiantseva et al 2014 |
| AK55 | - | Kazakhstan | *M.falcata* | Roumiantseva et al 2014 |
| AK57 | - | Kazakhstan | *M.falcata* | Roumiantseva et al 2014 |
| AK58 | - | Kazakhstan | *M.falcata* | Roumiantseva et al 2014 |
| AK62 | - | Kazakhstan | *M. trautvetteri* | Roumiantseva et al 2014 |
| AK64 | - | Kazakhstan | *M.falcata* | Roumiantseva et al 2014 |
| AK68 | - | Kazakhstan | *M.falcata* | Roumiantseva et al 2014 |
| AK70 | - | Kazakhstan | *M.lupulina* | Roumiantseva et al 2014 |
| AK75 | + | Kazakhstan | *M.lupulina* | Roumiantseva et al 2014 |
| AK83 | + | Kazakhstan | *M.falcata* | Roumiantseva et al 2014 |
| AK86 | - | Kazakhstan | *M.lupulina* | Roumiantseva et al 2014 |
| AK87 | + | Kazakhstan | *M.lupulina* | Roumiantseva et al 2014 |
| AK88 | - | Kazakhstan | *M.lupulina* | Roumiantseva et al 2014 |
| AK89 | - | Kazakhstan | *M.lupulina* | Roumiantseva et al 2014 |
| AK91 | - | Kazakhstan | *Melilotus sp.* | Roumiantseva et al 2014 |
| AK97 | - | Kazakhstan | *M. varia* | Roumiantseva et al 2014 |
| AK98 | - | Kazakhstan | *M. trautvetteri* | Roumiantseva et al 2014 |
| AL568G | + | Italy | *M. sativa* | Carelli et al. 2000 |
| AL703 | - | Italy | *M. sativa* | Carelli et al. 2000 |
| AL703GG | + | Italy | *M. sativa* | Carelli et al. 2000 |
| AL703H | + | Italy | *M. sativa* | Carelli et al. 2000 |
| AL703HH | - | Italy | *M. sativa* | Carelli et al. 2000 |
| AO641M | + | Italy | *M. sativa* | Carelli et al. 2000 |
| AO643DD | + | Italy | *M. sativa* | Carelli et al. 2000 |
| AO650BB | + | Italy | *M. sativa* | Carelli et al. 2000 |
| AO650BB | - | Italy | *M. sativa* | Carelli et al. 2000 |
| BE129GG | - | Italy | *M. sativa* | Carelli et al. 2000 |
| BE31LL | - | Italy | *M. sativa* | Carelli et al. 2000 |
| BE77GG | + | Italy | *M. sativa* | Carelli et al. 2000 |
| BL139L | - | Italy | *M. sativa* | Carelli et al. 2000 |
| BL224H | - | Italy | *M. sativa* | Carelli et al. 2000 |
| BL224L | + | Italy | *M. sativa* | Carelli et al. 2000 |
| BL225C | + | Italy | *M. sativa* | Carelli et al. 2000 |
| BL225L | - | Italy | *M. sativa* | Carelli et al. 2000 |
| BO112EF | - | Italy | *M. sativa* | Carelli et al. 2000 |
| BO114B | - | Italy | *M. sativa* | Carelli et al. 2000 |
| BO114E | - | Italy | *M. sativa* | Carelli et al. 2000 |
| BO122EE | - | Italy | *M. sativa* | Carelli et al. 2000 |
| BO197E | - | Italy | *M. sativa* | Carelli et al. 2000 |
| BO2166 | - | Italy | *M. sativa* | Carelli et al. 2000 |
| BO21CC | + | Italy | *M. sativa* | Carelli et al. 2000 |
| CE316II | + | Italy | *M. sativa* | Carelli et al. 2000 |
| CE367L | + | Italy | *M. sativa* | Carelli et al. 2000 |
| CE480L | - | Italy | *M. sativa* | Carelli et al. 2000 |
| CE480L (1) | + | Italy | *M. sativa* | Carelli et al. 2000 |
| CE480L (2) | + | Italy | *M. sativa* | Carelli et al. 2000 |
| CE480LL | + | Italy | *M. sativa* | Carelli et al. 2000 |
| CL374FF | - | Italy | *M. sativa* | Carelli et al. 2000 |
| CL375B | + | Italy | *M. sativa* | Carelli et al. 2000 |
| CL375B | + | Italy | *M. sativa* | Carelli et al. 2000 |
| CL375B | - | Italy | *M. sativa* | Carelli et al. 2000 |
| CL375B | - | Italy | *M. sativa* | Carelli et al. 2000 |
| CO401C | - | Italy | *M. sativa* | Carelli et al. 2000 |
| CO409LL | + | Italy | *M. sativa* | Carelli et al. 2000 |
| CO431A | - | Italy | *M. sativa* | Carelli et al. 2000 |
| CO438LL | + | Italy | *M. sativa* | Carelli et al. 2000 |
| IB53 | - | Iran | *M. sativa* | Talebi Bedaf et al. 2037 |
| SA23 | - | Tunisia | *M. truncatula* | Trabelsi et al. 2010 |
| SA52 | - | Tunisia | *M. truncatula* | Trabelsi et al. 2010 |
| SA56 | - | Tunisia | *M. truncatula* | Trabelsi et al. 2010 |
| SA57 | - | Tunisia | *M. truncatula* | Trabelsi et al. 2010 |
| SM15 | - | Tunisia | *M. truncatula* | Trabelsi et al. 2010 |
| SM16 | - | Tunisia | *M. truncatula* | Trabelsi et al. 2010 |
| SM18 | - | Tunisia | *M. truncatula* | Trabelsi et al. 2010 |
| SS22 | - | Tunisia | *M. truncatula* | Trabelsi et al. 2010 |
| SS23 | - | Tunisia | *M. truncatula* | Trabelsi et al. 2010 |
| SS24 | - | Tunisia | *M. truncatula* | Trabelsi et al. 2010 |
| SS49 | - | Tunisia | *M. truncatula* | Trabelsi et al. 2010 |

## Supplementary Table S2

# Supplementary Table S2. ACC deaminase activity. The ACC deaminase activity is reported. The average values ± standard deviation of three replicate experiments are reported.

|  | **AK83 (BM493)** | **1021 pSRK (BM261)** | **1021 + pSRK- *acdS* AK83 (BM641)** |
| --- | --- | --- | --- |
| ACC deaminase activity (µM alphaketoglutarate/mg protein/h) | 4.35 ± 0.04 | 1.69 ± 0.03 | 5.23 ± 0.05 |

## Supplementary Table S3

**Supplementary Table S3. Growth on VVM (Vincent Minimal Medium) with formamide as sole nitrogen source.** Data indicate average optical density (OD_600nm_) ± standard deviation from three replicate experiments of cultures grown for 20h from a starting inoculum at OD_600nm_=0.2. BM641 (*S. meliloti* 1021 + pSRK- *acdS* AK83) and BM261 (*S. meliloti* 1021 pSRK) strains.

|  | **BM641** | **BM261** |
| --- | --- | --- |
| **VMM without nitrogen** | 0.36±0.01 | 0.41±0.01 |
| **VMM+NH_4_Cl** | 0.96±0.03 | 0.73±0.02 |
| **VMM+ Formamide 30mM** | 0.88±0.02 | 0.69±0.02 |
| **VMM+ Formamide 20mM** | 0.61±0.02 | 0.45±0.02 |
| **VMM+ Formamide 10mM** | 0.38±0.01 | 0.33±0.02 |
| **VMM+ Formamide 5mM** | 0.35±0.01 | 0.27±0.02 |
